# Augmented Reality Based Navigation for Computer Assisted Hip Resurfacing: A Proof of Concept Study

**DOI:** 10.1007/s10439-018-2055-1

**Published:** 2018-05-23

**Authors:** He Liu, Edouard Auvinet, Joshua Giles, Ferdinando Rodriguez y Baena

**Affiliations:** 1 Mechatronics in Medicine Laboratory, Imperial College, London, UK; 2 MSk Lab, Imperial College, London, UK; 3 Department of Mechanical Engineering, University of Victoria, Victoria, BC Canada

**Keywords:** Computer assisted orthopedic surgery, Guide hole drilling, Surgical accuracy, Depth image registration, Natural workflow

## Abstract

Implantation accuracy has a great impact on the outcomes of hip resurfacing such as recovery of hip function. Computer assisted orthopedic surgery has demonstrated clear advantages for the patients, with improved placement accuracy and fewer outliers, but the intrusiveness, cost, and added complexity have limited its widespread adoption. To provide seamless computer assistance with improved immersion and a more natural surgical workflow, we propose an augmented-reality (AR) based navigation system for hip resurfacing. The operative femur is registered by processing depth information from the surgical site with a commercial depth camera. By coupling depth data with robotic assistance, obstacles that may obstruct the femur can be tracked and avoided automatically to reduce the chance of disruption to the surgical workflow. Using the registration result and the pre-operative plan, intra-operative surgical guidance is provided through a commercial AR headset so that the user can perform the operation without additional physical guides. To assess the accuracy of the navigation system, experiments of guide hole drilling were performed on femur phantoms. The position and orientation of the drilled holes were compared with the pre-operative plan, and the mean errors were found to be approximately 2 mm and 2°, results which are in line with commercial computer assisted orthopedic systems today.

## Introduction

Hip replacement has been proven to be an effective procedure for end-stage hip joint problems like severe osteoarthritis. Of all hip replacement procedures, hip resurfacing can sometimes be a preferable alternative for young and active patients due to the potential advantages of improved bone preservation, lower risk of hip dislocation, easier revision to total hip replacement,^13^ and encouraging functional outcomes. Like total hip replacement, hip resurfacing also contains operations on the femur and the acetabulum to remove the damaged bone and cartilage, after which femoral and acetabular components are placed to reconstruct the articular surface. In conventional femoral preparation, a specially designed mechanical jig is used to identify the axis of the femoral neck, then a guide wire is inserted along the axis so that subsequent reamers and cutters can be properly aligned with the femoral neck in order to achieve ideal component positioning and fit. Therefore, creating the central guiding axis through the femoral neck is crucial to the success of this surgical technique.

As with all joint replacements, implantation accuracy, which is directly related to the accuracy of the implantation technique and technology used, is one of the most important considerations as it significantly impacts joint functional recovery and longevity of the replacement.^9^,^28^ Studies have shown that varus malpositioning of the femoral component with a stem-shaft angle ≤ 130° can lead to higher risk of early complications such as femoral neck fracture.^4^ Although the implantation positional accuracy has not been reported to negatively affect clinical outcomes, inaccurate positioning will require larger femoral/acetabular components to avoid notching, resulting in less acetabular bone stock which can harm joint stability.^12^ Currently, bone resection and implant positioning are mainly achieved manually by the surgeon with the assistance of mechanical jigs; thus, the accuracy relies heavily on the surgeon’s skills and experience, which can take significant time to develop.^18^

To provide more effective assistance that improves surgeon accuracy, computer navigation can be introduced into the procedure. Conventional computer navigation displays information and guidance through two-dimensional (2D) monitors, which is not intuitive and may lead to eye–hand coordination problems and loss of depth perception.^1^,^16^ In contrast, augmented reality (AR) has the ability to overcome these challenges by overlaying three-dimensional (3D) information *in situ*, which has shown potential for intra-operative navigation.^6^ In contrast to virtual reality, which creates a completely virtual environment to the exclusion of the real world, AR does not block the real environment, but rather overlays the virtual information on it so that intuitive guidance is provided while the original task is not obscured. Therefore, in sensitive applications like surgery, AR could be a better choice to provide both safety and efficiency.^25^,^27^ In recent years, the development of AR headsets has made AR technology available for a wide range of tasks, and the Microsoft HoloLens (Microsoft, Inc.) is an outstanding example. As an optical see-through, self-contained holographic computer, HoloLens achieves self-tracking and information rendering together without any other auxiliary equipment, which greatly simplifies its setup and facilitates its integration into the operating room.

Apart from using optical see-through headsets, AR can be implemented in a variety of ways. The most basic one is monitor-based, where the surgical scene is displayed on a monitor with the augmenting information aligned and overlaid on it. This rudimentary approach still suffers from the eye–hand coordination problems that affect conventional surgical navigation.^6^,^25^ In order to improve the display of AR *in situ*, portable monitors like smart phones and tablets have more recently been used to capture the scene, augment it and display it to the user at the same time. However, due to limitations in processing power and the need to hold the display during use, their application remains limited to simple cases.^2^,^21^ Video see-through headsets are, in essence, monitor based AR devices with two augmented video streams displayed right in front of the eyes, which enable the inclusion of 3D perception. However, this advantage is compromised by the safety issue that once the headset stops working, the users will completely lose sight of what they are performing, which can be catastrophic during surgery.^24^ Other popular AR approaches include projecting virtual information directly on the real environment and overlaying information on a semi-transparent mirror. Although these approaches preserve the reality, the imbalance of overlaying 2D information onto a 3D scene may lead to lower accuracy and awkward ergonomics.^16^,^25^ As an upgrade from video see-through headsets and semi-transparent mirrors, optical see-through headsets such as the HoloLens possess many advantages from the approaches described earlier, such as portability, 3D *in situ* rendering, and reality preservation.^26^,^29^ Admittedly, problems still exist. Accuracy and latency issues are limited by the computing and sensing abilities, occlusion is almost inevitable, and the rendering techniques are still basic, resulting in unnatural interactions with the virtual objects. However, with the expected improvements in sensor technology, registration algorithms, and medical image processing arising from improved manufacturing methods, cloud computing and machine learning, optical see-through headsets have the potential to eventually become ubiquitous, in medicine and elsewhere.

Consequently, the aim of our study is to explore the possibility of applying AR based 3D computer navigation to hip resurfacing, and more specifically, to femoral preparation. Since most of the instruments for femoral preparation are based around the premise of creating a central guiding hole along the axis of the femoral neck, in this paper, our goal is to simplify the procedure of locating and drilling such a hole by means of optical see-through AR assistance and an automatic registration and limb tracking system. We employ *in vitro* testing with a small user group to assess both the accuracy and repeatability of guide hole drilling with our setup.

## Materials and Methods

In this paper, we developed a navigation system based on depth sensing technology and the HoloLens. The AR guidance was generated according to a pre-operative plan, which was shown to the user through the HoloLens. In order to accurately display the guidance information in the HoloLens display, our previously developed robotic registration system^14^ was used to measure the pose of the target femur.

### Previous Camera–Robot Registration System

**Figure 1:**
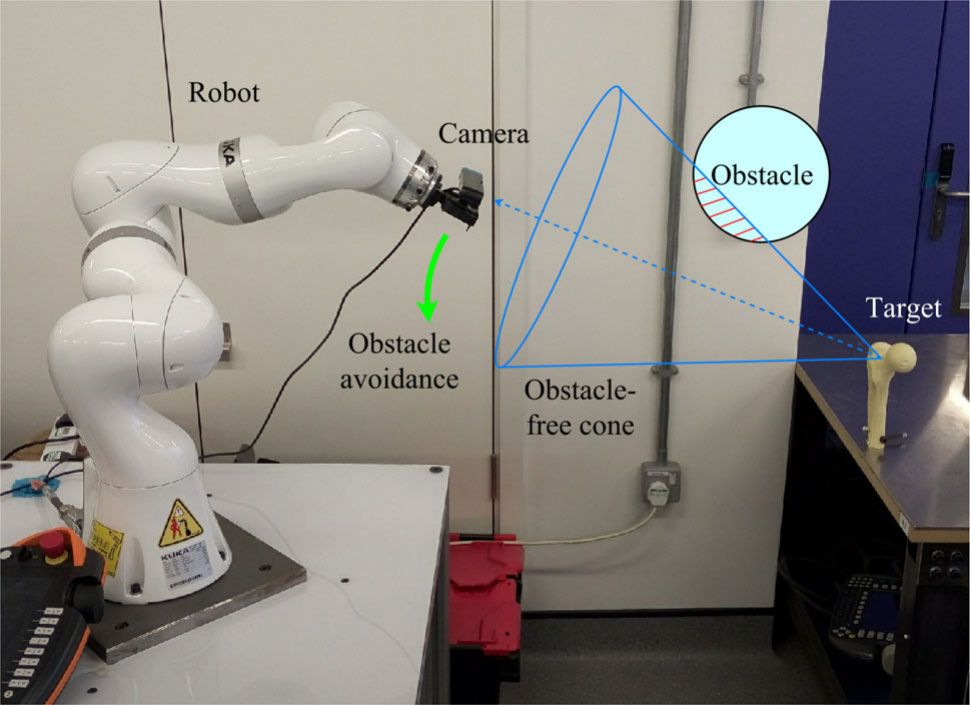
The camera–robot registration system with obstacle avoidance.

At the basis of any navigation system is the need to correctly register the geometry/s of interest, as this process is integral to locating and tracking objects within the surgical scene. In this paper, the registration object is the femur. Instead of using optical markers, our registration system is built on a depth camera with the assistance of robotic technology, as shown in Fig. 1, with technical details about the complete setup available in our previous open access publication.^14^ The depth camera (Xtion Pro Live, Asustek Computer, Inc., Taipei, Taiwan, China), which has the ability to capture the environment’s geometry in the form of a 3D point cloud, is mounted on, and calibrated to, the end-effector of a serial manipulator (LBR iiwa, KUKA Aktiengesellschaft, Augsburg, Bavaria, Germany). The point cloud of the environment is processed to identify descriptors of the geometrical features, which are then compared with descriptors of the pre-scanned femur model to obtain a rough estimate of the femur pose. This gross registration step is then refined by a standard implementation of the iterative closest point (ICP) algorithm^5^ so that the femur pose can be ascertained with accuracy, to enable surgical navigation. Once the first ICP succeeds, the registration result of this iteration will be used as the initial estimate for the subsequent one. This process runs continuously at approximately 10 Hz in order to track the target femur dynamically during the procedure. After the target is located, a conical region is defined based on a line connecting the target and the camera, and objects that are detected by the depth camera to be located inside the cone are tracked as obstacles. The robot will move the camera to avoid occlusion while keeping the target at the center of the camera view. Meanwhile, admittance control is applied to the robot so that direct manipulation by human operators is allowed, thus making the system more user-friendly and intuitive.

While clinical deployment of robotic-based obstacle avoidance would require further consideration (autonomous robotic motion would have to negotiate the crowded operating theater space), in principle it would address the line-of-sight problem that exists in optical tracking and reduce the possibility of tracking failure. These advantages would give more freedom to the surgeon and alleviate the mental burden caused by the continual need to adjust traditional optical navigation systems to avoid occlusion, if an appropriate and safe implementation of the system could be provided. Consequently, our robot-based registration and tracking system was employed in this work.

### AR System Setup and Workflow Control

The AR system includes four parts: the depth camera, the robot, the HoloLens, and a tracked surgical drill. Each of these has its own coordinate system, and appropriately establishing a correspondence between them is the key to accurate AR performance. The calibration between the depth camera and the robot end effector has been described in our previous study.^14^ After registration, the target femur geometry is described in the depth camera coordinate frame. In order for the HoloLens to use this data, the transformation from the depth camera frame to the HoloLens frame must be found. To do this, we use an image marker (*marker1*) which can be recognized by the RGB cameras on both the depth camera and the HoloLens. Its pose measured by both cameras can be denoted by two 4 × 4 time-varying matrices, $T_{{{\text{marker}}1}}^{\text{cam}} (t)$ and $T_{{{\text{marker}}1}}^{\text{holo}} (t).$ Thus, the correspondence between the two camera frames is obtained, and the target pose can be transformed into the HoloLens frame by

1 $$T_{\text{target}}^{\text{holo}} (t) = T_{{{\text{marker}}1}}^{\text{holo}} (t) \times \left( {T_{{{\text{marker}}1}}^{\text{cam}} (t)} \right)^{ - 1} \times T_{\text{target}}^{\text{cam}} (t),$$

where $T_{\text{target}}^{\text{holo}} (t)$ and $T_{\text{target}}^{\text{cam}} (t)$ are the target poses with respect to the HoloLens and the depth camera, respectively.

According to $T_{\text{target}}^{\text{holo}} (t),$ a virtual femur model with the corresponding navigation information can be rendered for the user. However, updating $T_{\text{target}}^{\text{holo}} (t)$ through time by Eq. (1) requires *marker1* to remain in view at all times, which is impractical. In order not to present *marker1* all the time, at *t* = 0, the pose of the rendered femur model in world frame $T_{\text{target}}^{\text{world}} (0)$ [which is computed from $T_{\text{target}}^{\text{holo}} (0)$] is recorded as a proprietary HoloLens “spatial anchor” [i.e., anchor = target(0)], such that subsequent target pose updates are computed relative to this anchor [i.e., $T_{\text{target}}^{\text{anchor}} (t)$]. Since the robot base frame and the anchor frame are both static relative to the world frame, obviously, we have

2 $$T_{\text{cam}}^{\text{base}} (0) \times T_{\text{anchor}}^{\text{cam}} (0) \times T_{\text{target}}^{\text{anchor}} (t) = T_{\text{cam}}^{\text{base}} (t) \times T_{\text{target}}^{\text{cam}} (t),$$

where $T_{\text{cam}}^{\text{base}} (0)$ and $T_{\text{anchor}}^{\text{cam}} (0)$ are constant matrices recorded at *t* = 0, and $T_{\text{cam}}^{\text{base}} (t)$ is calculated from the forward kinematics of the robot.

From Eq. (2), we can have the real-time update of the target pose

3 $$T_{\text{target}}^{\text{anchor}} (t) = \left( {T_{\text{anchor}}^{\text{cam}} (0)} \right)^{ - 1} \times \left( {T_{\text{cam}}^{\text{base}} (0)} \right)^{ - 1} \times T_{\text{cam}}^{\text{base}} (t) \times T_{\text{target}}^{\text{cam}} (t).$$

We can see from Eq. (3) that the rendered target is updated continuously without the need for additional markers, as long as the target is tracked by the depth camera and the robot kinematics is computed.

Another image marker, *marker2*, is used and fixed to the robot base in order to align the HoloLens with the robot before registration is carried out, and the transformation from *marker2* to the robot base, $T_{\text{marker2}}^{\text{base}} ,$ is constant and known by the time the system is set up. Once *marker2* is recognized by the HoloLens, the correspondence between the depth camera and the HoloLens is established as follows

4 $$T_{\text{holo}}^{\text{cam}} (t) = T_{\text{base}}^{\text{cam}} (t) \times T_{{{\text{marker}}2}}^{\text{base}} \times \left( {T_{{{\text{marker}}2}}^{\text{holo}} (t)} \right)^{ - 1} ,$$

where $T_{\text{holo}}^{\text{cam}} (t)$ is the transformation matrix from the HoloLens to the depth camera, $T_{\text{base}}^{\text{cam}} (t)$ is the inverse of $T_{\text{cam}}^{\text{base}} (t),$ and $T_{\text{marker2}}^{\text{holo}} (t)$ is the pose of *marker2* in the HoloLens frame.

**Figure 2:**
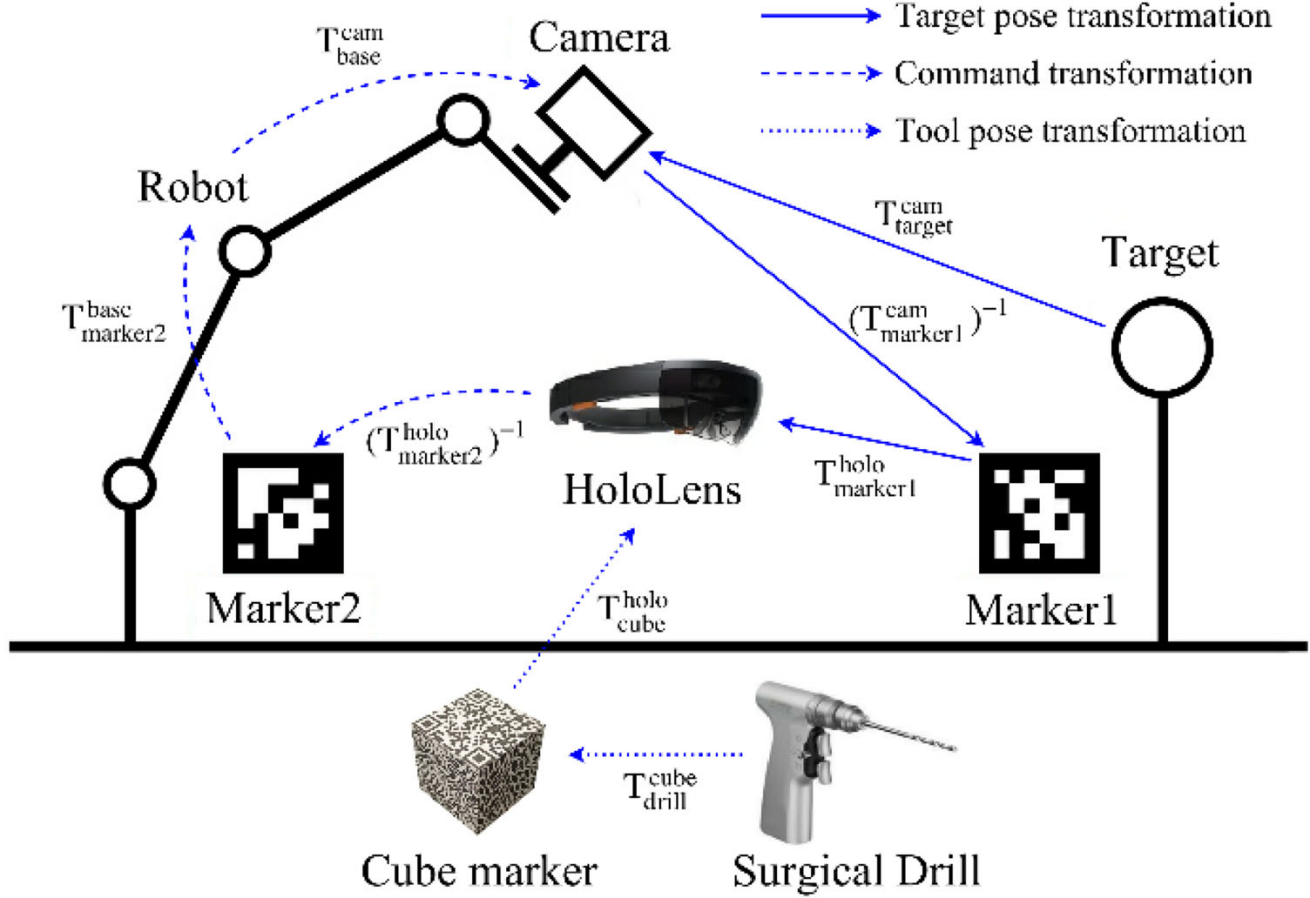
Relationships between coordinates of different components of the AR navigation system.

As for the surgical drill, a cube shaped marker that can be tracked by the HoloLens is fixed onto it. After calibration between the drill and the marker, the coordinates of the tip and the axis of the drill are known in the cube marker frame (i.e., $T_{\text{drill}}^{\text{cube}}$ is constant and known). These coordinates will also be transformed into the HoloLens frame by

5 $$T_{\text{drill}}^{\text{holo}} (t) = T_{\text{cube}}^{\text{holo}} (t) \times T_{\text{drill}}^{\text{cube}} ,$$

where $T_{\text{cube}}^{\text{holo}} (t)$ is the cube marker pose tracked by the HoloLens. The drill will then be compared with the transformed pose of the target femur. If, after calculation, the drill is properly aligned with the planned guide hole, the HoloLens will inform the user of the results so that the user can proceed with drilling. All relationships between each of the coordinate systems composing the whole system are shown in Fig. 2.

To minimize the learning burden on the user, interaction with the system is achieved through intuitive methods such as voice commands, gestures, and manual manipulation. These human inputs are interpreted automatically by the HoloLens or the robot so that no additional control devices are necessary. The AR integrated workflow is divided into several stages by simple voice commands, with different tasks performed in each stage:

1. After the system is started, the robot is force compliant so that the surgeon can set its configuration by hand to make the target roughly in view of the depth camera.
2. The surgeon wearing the HoloLens defines the HoloLens and robot correspondence by looking at *marker2* to obtain the matrix $T_{\text{marker2}}^{\text{holo}} (t).$
3. With the help of HoloLens gaze and gesture features, a spherical ROI that contains the target femur is selected by the surgeon with respect to the HoloLens frame, then the ROI position is transformed into the depth camera frame using Eq. (4) and sent to the registration controller. This ROI guides where the depth camera should look, and the point cloud outside the ROI will not be processed, thus increasing registration speed.
4. The point cloud inside the ROI is processed to register the target femur in the depth camera frame and estimate the target pose $T_{\text{target}}^{\text{cam}} (t).$
5. *Marker1* is provided for the depth camera and HoloLens to acquire $T_{\text{marker1}}^{\text{cam}} (t)$ and $T_{\text{marker1}}^{\text{holo}} (t)$ so that the target pose is available for HoloLens using Eq. (1) and the 3D navigation can be rendered, then updated by Eq. (3)
6. The surgeon holds the surgical drill and registers it to the HoloLens, then follows the navigation cues to perform the planned drilling.

**Figure 3:**
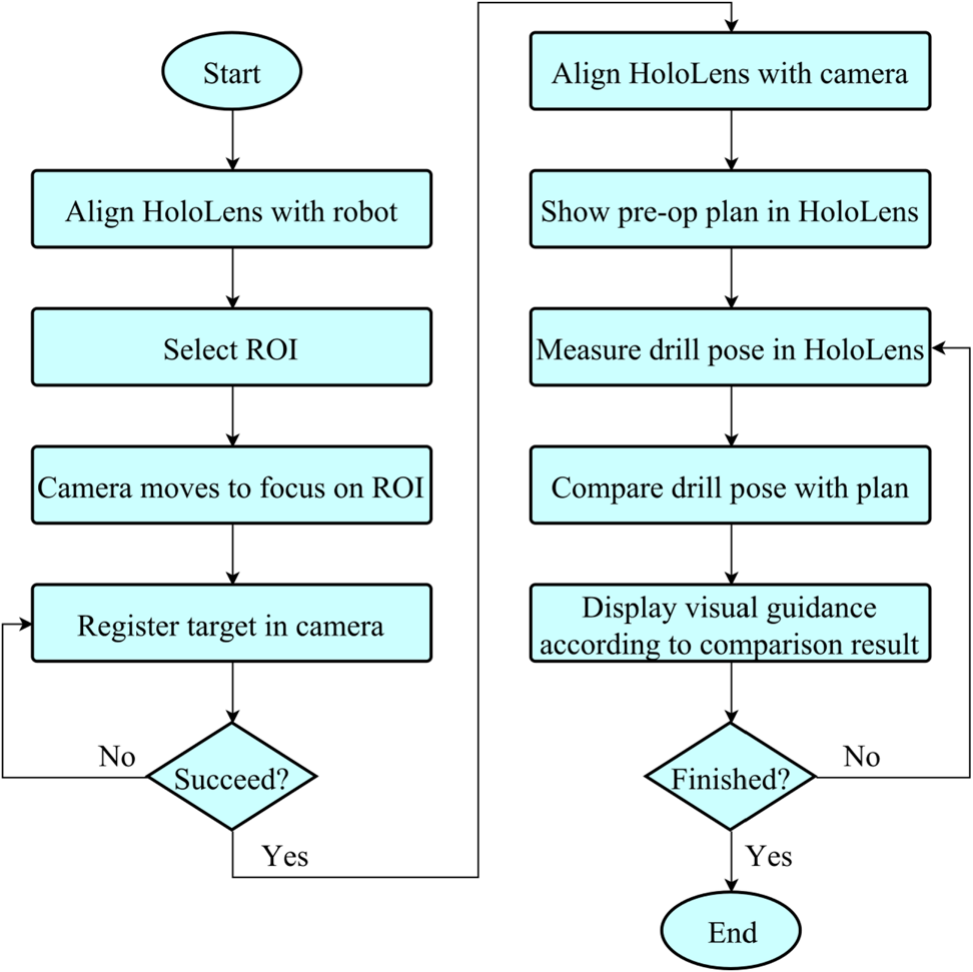
AR guided workflow for guide hole drilling.

The complete workflow for drilling of the guide hole into the femur with AR navigation is shown in Fig. 3.

### Experiment Design

**Figure 4:**
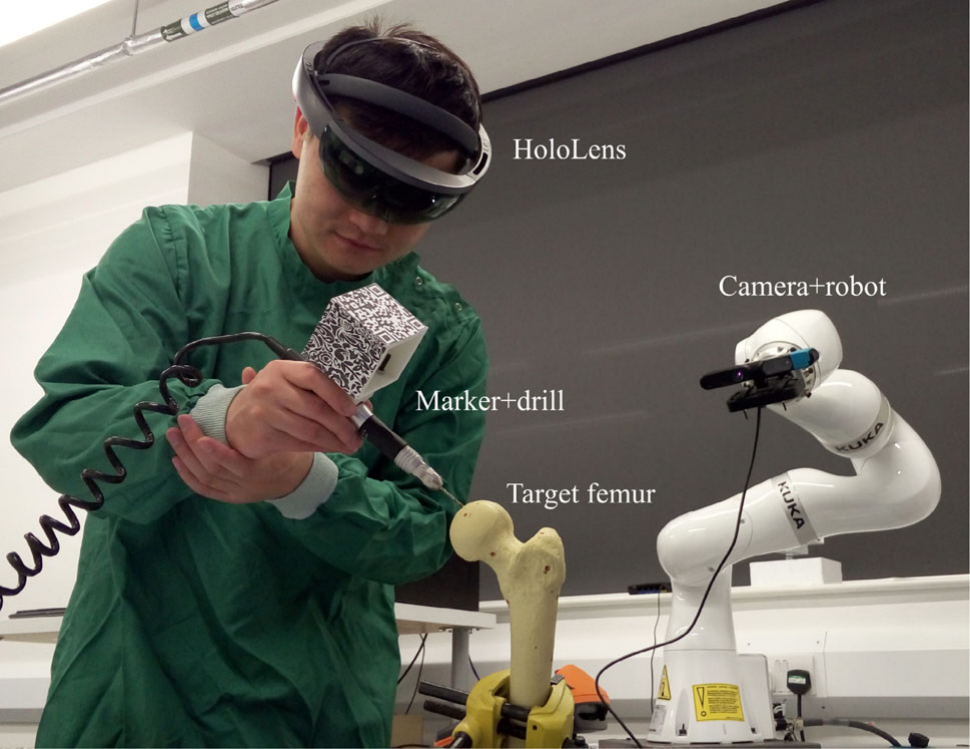
Actual use of the AR navigation system to assist guide hole drilling.

Experiments were performed to test the accuracy of the proposed AR navigation system. In this paper, we apply the system to the hip resurfacing procedural step of guide hole drilling. Instead of requiring a mechanical alignment jig to locate the axis of the femoral neck, AR navigation provides visual guidance overlaid onto the surgeon’s view, where the approach vector for the guide hole is rendered *in situ*, such that drilling can be performed free-hand and without having to look away from the patient. Three groups of experiments were carried out under different conditions, each consisting of 30 trials. The first group was performed by the author to test the repeatability of the system. The second group was also performed by the author, but disturbances were introduced during the procedure (e.g., obstacles appeared between the camera and the target femur, leading to obstacle avoidance motion of the robot). In the third group, five students without medical background volunteered to perform six trials each, and before the experiments they were trained to use the whole system for about an hour. The actual setup for the experiment of guide hole drilling is shown in Fig. 4.

**Figure 5:**
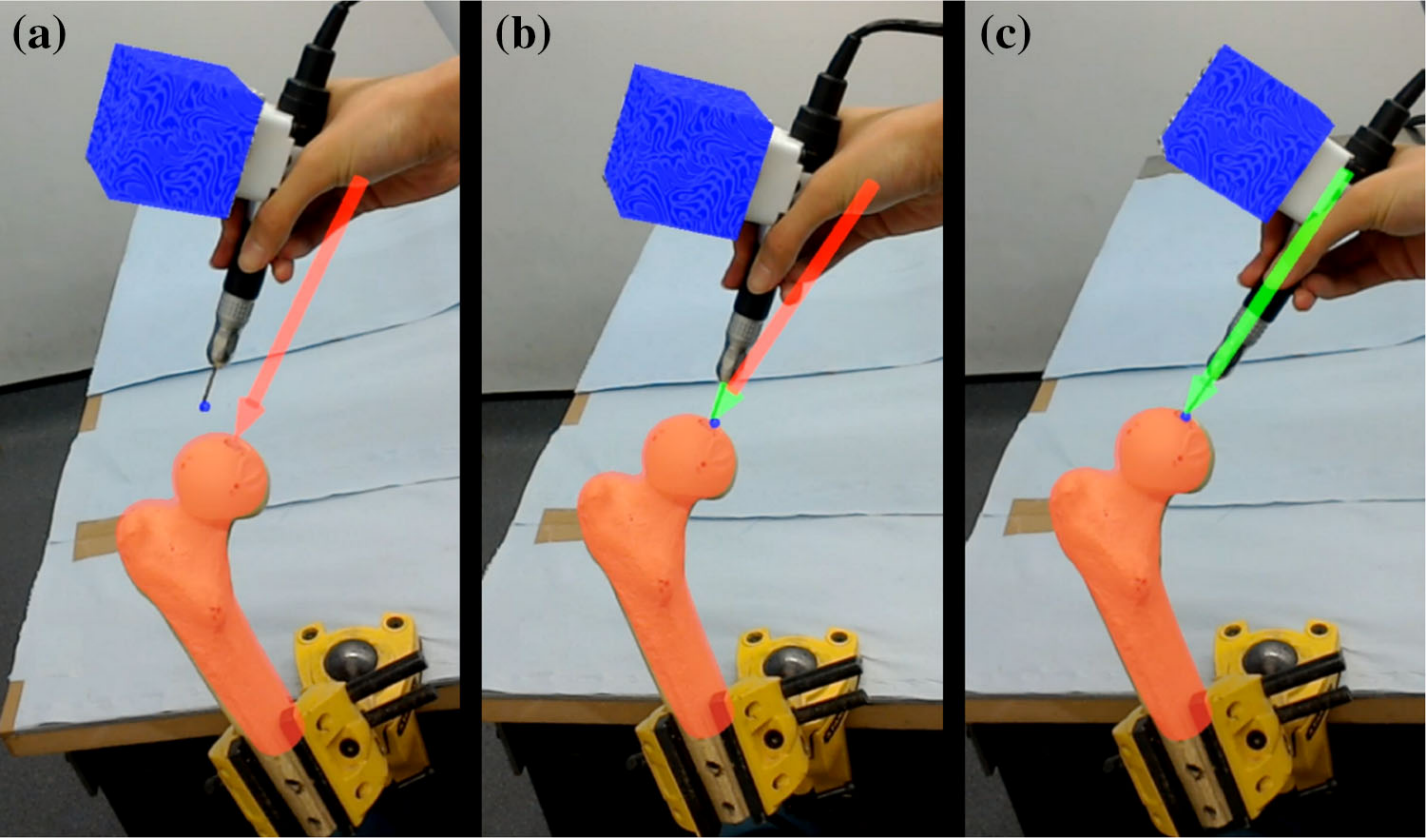
Visual guidance through HoloLens for guide hole drilling. This was recorded from the user’s view. (a) Inaccurate position and direction. (b) Accurate position but inaccurate direction. (c) Accurate position and direction.

The experiments were performed on foam femur phantoms (SKU 1129, Sawbones, Pacific Research Laboratories, Vashon, Washington, US), the surface geometry of which was 3D scanned beforehand to obtain the anatomical model used for the pre-operative plan and the model-based registration. After the target was registered in the HoloLens, a virtual red arrow was shown to the user, indicating the planned entry point and the drilling direction. Once the drill was also registered in the HoloLens frame, its position and orientation were compared with the arrow, and the arrow head and shaft turning green indicated that the position difference and the orientation difference were within the thresholds, respectively (in the experiments, the position error threshold was set to 3 mm and the orientation threshold was 2.5°). When the whole arrow was green, the user could proceed with drilling. The HoloLens guidance feedback is shown in Fig. 5. It was ensured that the arrow was kept green during drilling.

Ten fiducial points with known positions on the scanned femur model were used as the gold standard measurements for comparison. After drilling, the position and direction of the guide hole were measured together with the positions of the 10 fiducial points using a MicroScribe (G2X, Solution Technologies, Inc., Oella, Maryland, US), then a closed form, paired point registration method^11^ was used to calculate the transformation from the measured fiducial positions to the fiducial positions on the femur model. The measured position and direction of the guide hole could then be transformed to the femur model frame so that they could be compared with the pre-operative plan to obtain the errors. Time spent on each procedure was also recorded during the experiments. An analysis of variance (ANOVA) was adopted to compare the results of different volunteers, and Student’s *t*-tests were performed to compare results between groups.

An external PC running Ubuntu 16.04 was used to process the images from the depth camera, and to control the robot through KUKA’s Fast Robot Interface (KUKA Roboter GmbH, Augsburg, Bavaria, Germany). Image processing was based on the Point Cloud Library^23^ and the AprilTags C++ Library,^20^ while the Eigen Library^10^ was used to facilitate coding of the robotic controller. Unity (Unity Technologies SF, San Francisco, California, US), Vuforia (PTC, Inc., Needham, Massachusetts, US) and HoloToolkit (GitHub, Inc., San Francisco, California, US) were used to develop the program for the HoloLens. The communication between the HoloLens and the external PC was *via* the user datagram packet over internet protocol. Result processing was done in MATLAB (R2016a, MathWorks, Inc., Natick, Massachusetts, US), and the Statistical Package for the Social Sciences (SPSS, version 24, IBM, Armonk, New York, US) was used to conduct the statistical analysis.

## Results

The time cost of the whole procedure, including loading the program onto the HoloLens and the robot controller, selecting the ROI, registering the target femur with the depth camera, rendering AR navigation *via* the HoloLens, and drilling the guide hole with AR navigation, was recorded as a reference. The average time was about 2 min (115 s) for the author and 4 min (234 s) for other users. With more practice, all the users were able to finish the procedure within 3 min.

**Figure 6:**
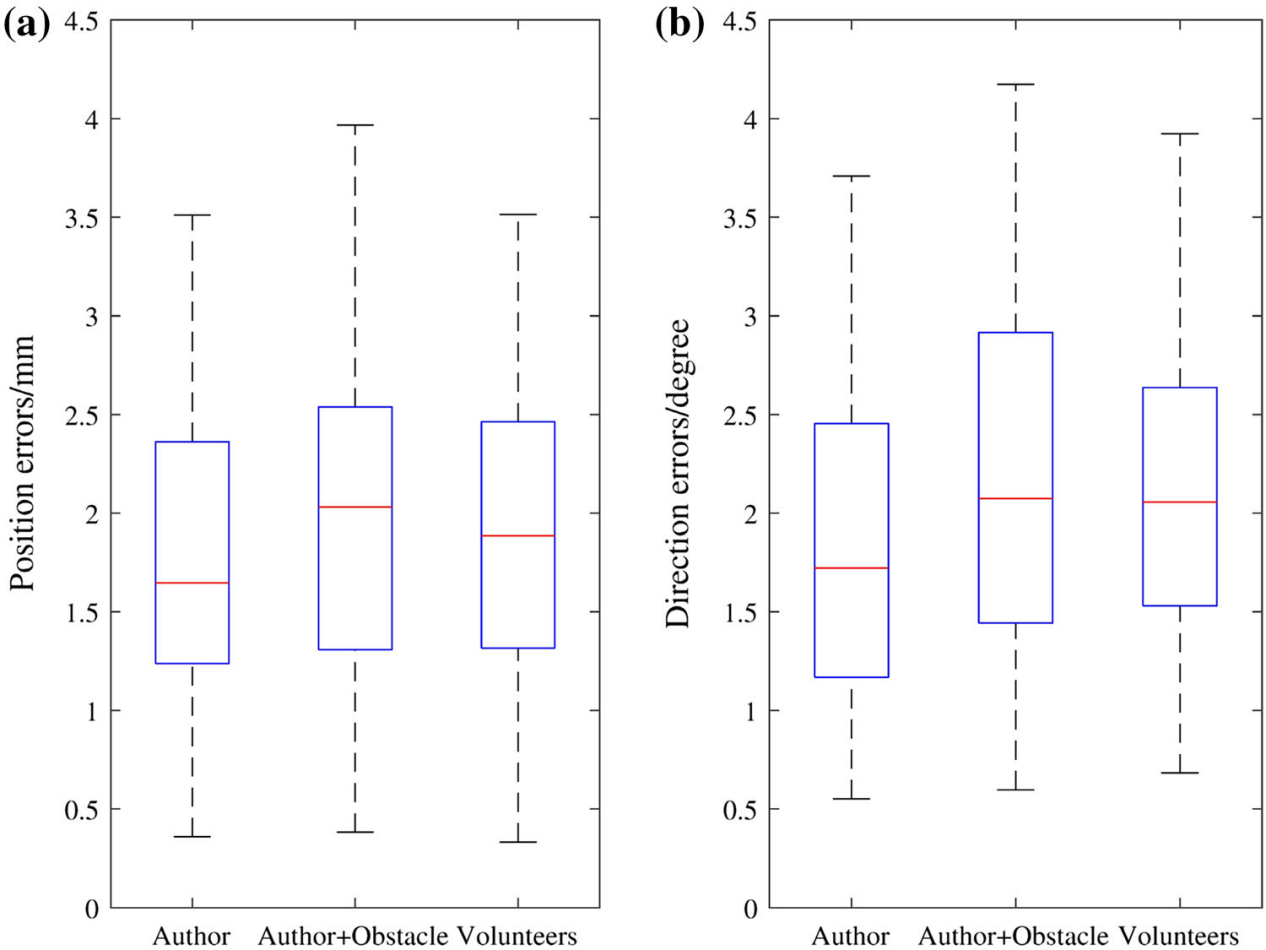
Box-and-whisker plots of the absolute errors in position and direction. (a) Position errors of the entry points. (b) Direction errors of the guide holes. The central mark indicates the median, and the bottom and top edges of the box indicate the 25th and 75th percentiles, respectively. The whiskers extend to the most extreme data points not considered outliers (approximately ± 2.7*σ* and 99.3% coverage for a normally distributed dataset), while outliers are plotted individually using the ‘+’ symbol.

**Figure 7:**
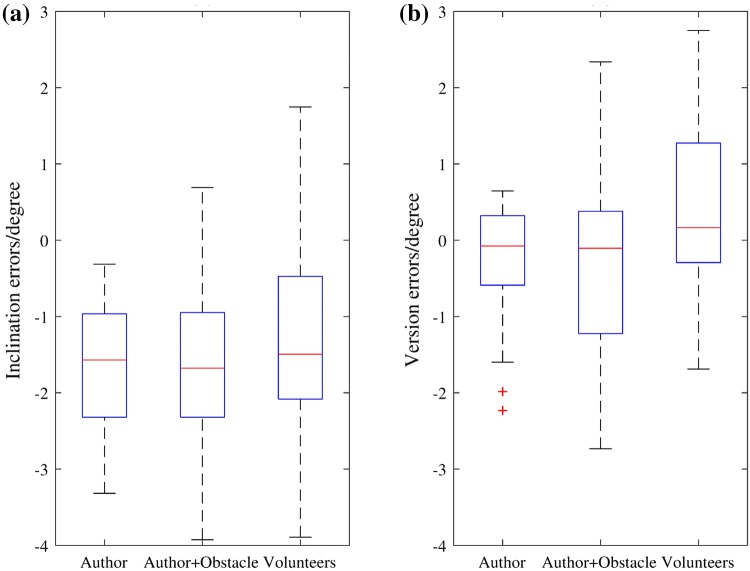
Box-and-whisker plots of the inclination errors and the version errors of the guide holes. (a) Inclination errors (positive values represent relative valgus and negative values represent relative varus). (b) Version errors (positive values represent anteversion and negative values represent retroversion).

**Figure 8:**
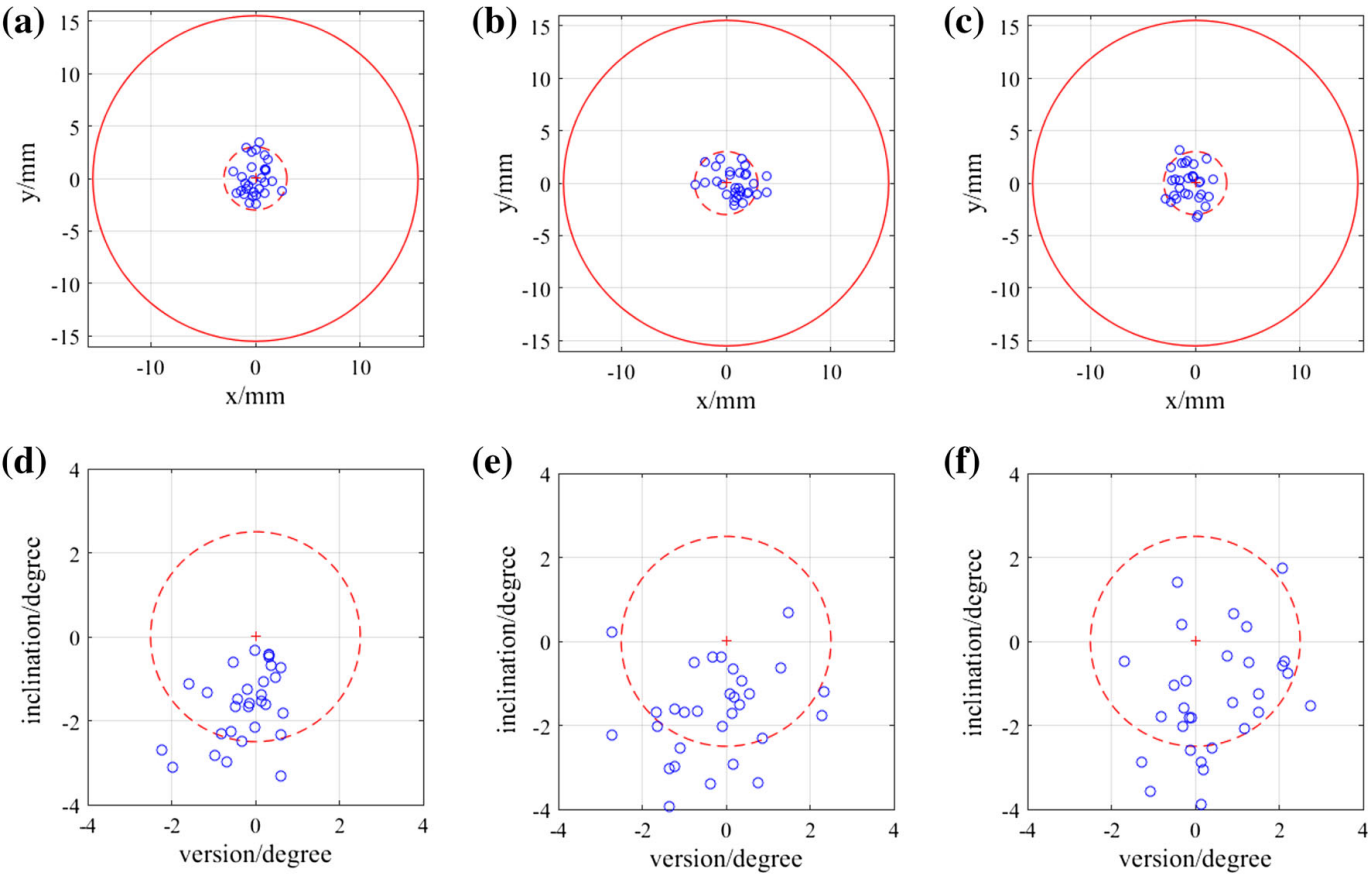
Scatter plots of the entry points and angular errors of the three groups. (a)–(c) Projections of the entry points of Author, Author + Obstacle and Volunteers groups on the plane perpendicular to the planned drilling direction. The origin is the planned entry point. The dashed circle is the positional threshold for AR guidance. The solid red circle represents the femoral neck diameter, which is 31 mm. (d)–(f) Inclination and version errors for Author, Author + Obstacle and Volunteers groups. The dashed circle is the directional threshold for AR guidance.

Position of the entry point on the femur phantom and direction of the guide hole were measured and transformed into the femur model frame to compare with the pre-operative plan. The angular errors were measured in 3D as absolute values, then represented in clinically relevant inclination and version errors. The mean values and standard deviations of the errors of each group are shown in Table 1. Figure 6 displays the variations of the absolute errors, and Fig. 7 displays the inclination errors and the version errors. Scatter plots of the entry position errors and direction errors of the measured guide holes are shown in Fig. 8.

**Table 1:** Errors in position and direction of the experiment results.

	Author	Author + Obstacle	Volunteers	Total
3D position error (mm)	1.76 ± 0.77	2.03 ± 0.87	1.91 ± 0.89	1.90 ± 0.84
3D direction error (°)	1.85 ± 0.89	2.20 ± 0.96	2.14 ± 0.81	2.06 ± 0.89
Inclination error (°)	− 1.62 ± 0.87	− 1.67 ± 1.10	− 1.30 ± 1.38	− 1.53 ± 1.14
Version error (°)	− 0.24 ± 0.76	− 0.24 ± 1.25	0.47 ± 1.14	0 ± 1.11

The errors are represented as mean ± standard deviation

Statistical analysis was conducted on the absolute errors of the three groups. One-way between subjects ANOVAs were used to compare the position and direction errors of the five volunteers in the Volunteers group, and no statistically significant differences were found in either position errors (*p* = 0.370) or direction errors (*p* = 0.900). The *t*-tests showed no significant differences between the Author group and the Volunteers group (position error: *p* = 0.493, direction error: *p* = 0.188), indicating good usability of the AR navigation system (i.e., with some practice, an inexperienced user can achieve similarly accurate results). The differences between the Author group and the Author + Obstacle group were also insignificant (position error: *p* = 0.200, direction error: *p* = 0.146), demonstrating appropriate dynamic tracking of the limb during the navigation of guide hole drilling.

## Discussion

In this study we tested the accuracy of drilling a guide hole along the axis of the femoral neck for hip resurfacing with our AR based navigation system. Under different conditions, the position and direction errors from the pre-operative plan were around 2 mm and 2°, results which are promising. Although we did not perform another set of experiments with other techniques to compare the outcomes, several studies have been done on the accuracy of guide wire insertion with conventional jigs, patient specific instrumentation (PSI) and navigation,^3^,^8^,^12^,^19^ and their results are briefly listed in Table 2. Since the measurements and experimental conditions are not the same, results cannot be compared directly. However, the mean errors of our experiments are comparable to those in these studies, and our standard deviations are generally smaller. According to the quantitative score table for guide concepts proposed by Audenaert *et al*.,^3^ the achieved accuracy is scored as ‘acceptable’ if the position error ≤ 4 mm and the direction error ≤ 4°, and as ‘good’ if the position error ≤ 2 mm and the direction error ≤ 2°. Therefore, the accuracy of this AR navigation system under the somewhat artificial test conditions described here is within the ‘acceptable’ range, and very close to the ‘good’ range.

**Table 2:** Results of previous studies on guide wire insertion for hip resurfacing.

Studies	Subjects	Methods	Positional error (mm)	Angular error (°)
Kitada *et al*.^12^*	Phantoms	Conventional jig	− 0.7 ± 6.0, 2.8 ± 4.0, − 1.1 ± 6.2	− 5.3 ± 10.6, 1.9 ± 5.0
		PSI	− 1.1 ± 1.1, 0.5 ± 1.6, 1.7 ± 2.5	2.4 ± 1.5, 1.8 ± 3.1
		CT-based navigation	− 0.8 ± 2.1, 1.2 ± 1.9, 1.7 ± 2.8	2.5 ± 2.4, 1.5 ± 2.3
Cobb *et al*.^8^^†^	Phantoms	Conventional jig	3 ± 3.4, 2 ± 4.4	4 ± 4, − 2 ± 5
		CT-based navigation	1 ± 1.6, 0 ± 1.1	1 ± 1, − 1 ± 2
		Imageless navigation	1 ± 6.2, 2 ± 2.1	0 ± 2, 0 ± 9
Olsen *et al*.^19^^†^	Phantoms	Imageless navigation	N/A	0.2 ± 1.5, − 4.2 ± 3.8
	Cadavers	Imageless navigation	N/A	1.1 ± 1.0, − 0.1 ± 2.3
Audenaert *et al*.^3^^‡^	Patients	PSI	2.73 ± 1.97	4.05 ± 1.84
Our study^‡^	Phantoms	AR navigation	1.90 ± 0.84	2.06 ± 0.89

Results are in the form of mean ± standard deviation

*Positional errors are measured in superior, medial and anterior directions, angular errors are measured for inclination and anteversion

^†^Positional errors are measured in posterior and inferior directions; angular errors are measured for inclination and anteversion

^‡^Positional and angular errors are measured in 3D

In addition to its accuracy and precision, the AR navigation system also shows promising advantages in terms of surgical workflow. With some simple voice commands and gestures, the user could easily interact with the HoloLens, through which the depth camera and the robot are controlled. In this way, a user-centered workflow is achieved, where all other equipment will serve the user rather than the user needing to operate the equipment. The registration is automatic and markerless, only requiring a model of the bone for pre-operative planning purposes, as is customary in image-based computer assisted orthopedic systems. Importantly, it is not necessary to insert bone markers or to manually select bone features to register the markers, which has the potential to reduce intra-operative time. Finally, this AR navigation technique also possesses usability advantages over conventional navigation on computer monitors by allowing intuitive visual guidance that does not distract from the surgical field. The system’s somewhat higher complexity in comparison to traditional navigation is warranted in this proof-of-concept setup, as it is shown to have the potential to provide a more informative surgical environment, which may eventually contribute to higher efficiency and better surgical accuracy. Significant improvements in the commercial offering for AR headsets and low-cost surgical robotic assistants are also expected to alleviate this issue in the future.

Current limitations of the system, as given in the feedback from the volunteers after the experiments, can be grouped into the following aspects:

- Depth perception of the directional guidance. Although the guidance arrow is rendered in 3D, it cannot be completely opaque to occlude objects behind it. And objects in front of the arrow cannot occlude it, either, as the arrow is projected to the eyes directly. This may cause difficulties in perceiving the depth information of the virtual object, so it takes some practice to align the real drill axis with the virtual arrow.
- Deviations of the HoloLens display for the user. HoloLens does not track the user’s eye positions (e.g., eye width), so for different users the position of the hologram may appear in a slightly different location within the immediate visual scene. Although this does not affect the actual position of the hologram, which is pinned to the scene in a pre-operatively defined pose, this projective distortion can be misleading for the user during AR-based navigation.
- The increased complexity of the system. Although the AR navigation system may help simplify the surgical workflow, the whole procedure may become technically more complex because of the use of different technologies such as robotics and an AR headset. Other techniques like voice commands and gaze control may also cause interruptions or even unexpected responses of the system when the commands are misinterpreted. This added complexity and the robustness issues could be address with further development (e.g., with a bespoke robotic effector, a ceiling mounted option, a multiple-camera setup, and less intrusive AR glasses), but represent significant limitations of this proof-of-concept work.
- Stability of free-hand drilling. Although none of our volunteers were medically trained, all found it difficult to maintain good stability during free-hand drilling [inclination errors are more varus for all the three groups (*p* < 0.05)], an issue which might require further investigation.

The biggest obstacle to eventual clinical deployment is registration in a realistic scenario. Specifically, the challenge will be to identify suitable image processing algorithms to segment the target femur from the surgical scene in order to correctly register it. In our study, the conditions are artificially simple, with a femur phantom isolated from the leg, no soft tissue or blood, and no drapes to contend with. Conventional feature-based segmentation may encounter significant difficulties in such a complex environment, but we believe that the exponential growth in machine learning techniques and technologies in recent years may eventually come to the rescue. We are currently exploring this line of research *via* the application of fully convolutional networks,^15^ which have shown promising results in 2D and 3D medical image registration,^7^,^17^,^22^ to our AR setup.
